# Analysis of the Literature Pertaining to the Education of Public Health Professionals

**DOI:** 10.3389/fpubh.2013.00047

**Published:** 2013-11-05

**Authors:** Connie J. Evashwick, Donghua Tao, Kate Bax

**Affiliations:** ^1^College of Health and Human Services, George Mason University, Fairfax, VA, USA; ^2^Medical Center Library, Saint Louis University, St. Louis, MO, USA

**Keywords:** education of the public health workforce, public health workforce pedagogy, literature on public health pedagogy, literature on education of public health professionals, public health professionals’ education

## Abstract

A well-educated workforce is essential to the infrastructure of a public health system (1). At a time when global focus on public health is increasing, a severe shortage of public health professionals is projected (2). A strong educational framework is thus imperative to ensure the capacity and capability of the worldwide public health workforce for the future. The education of those who work in public health is spread across disciplines, subject-specific training programs and types of academic institutions. In the 2011 report on the *Health Professionals for a New Century*, Frenk and Chen comment that, compared to medicine and nursing, public health has done the least to examine what and how it teaches (3). This does not bode well for meeting the demands of the public health workforce for the future. The purpose of the study reported here is to analyze the state of pedagogy pertaining to the education of the public health workforce as evidenced by published literature. The focus is on “professionals,” defined as those who have formal education, are self-governing, and can work independently.

## Introduction

The overarching goal of this study is to examine the literature pertaining to the education of public health professionals in recent years in order to document the extent to which those preparing professionals of the public health workforce think about what they teach, how they teach, and what the results of the instruction are. The specific objectives are to (1) identify relevant peer reviewed literature, (2) analyze that literature for content, (3) characterize the literature according to teaching elements, and (4) draw conclusions and implications to enhance future pedagogical efforts related to the education of public health professionals.

Identifying those who comprise the public health workforce can be a challenge, particularly if cross-national comparisons are involved. For the purposes of this study, “public health professional” is used to refer to professionals of any discipline working in any aspect of the field of public health, including in government and non-government sectors, on a full-time or part-time basis. “Professional” refers to characteristics that include formal academic training, the ability to work independently, a self-governing discipline, and adherence to a code of ethics (4). The study does not distinguish “practitioners,” often considered the front-line providers of public health, from academicians, policy-makers, administrators, or others. The focus of the study is on pedagogy for the training of professionals, and thereby does not include the many programs that train various types of support personnel, such as village health workers or data entry clerks tracking disease reporting.

“Pedagogy” is defined here as the study of the science of teaching, including content, format, compilation into curricula, and evaluation. Elements of pedagogy include learning objectives, KSAs (knowledge, skills, and attitudes), courses, curricula, competencies, instructional methods, and evaluation methodologies. Workforce analyses, needs assessments, and job placement outcomes are elements that affect education. “Accreditation” as defined by the US Department of Education is recognition granted by an authorized accrediting body that an educational program meets minimum standards specified by its field.

The challenges of identifying literature on the pedagogy of public health workforce education include the broadness of the field and the vagueness of the terms. “Public health education” can be interpreted to mean the education of the public about health. A general literature search turns up articles on anti-smoking campaigns and condom use. The breadth of the field means that literature on pedagogy can be found on such diverse topics as epidemiology, sanitary engineering, and nutrition. Several health professions disciplines have specialties of public health within their academic training, such as public health nurses and physicians with specialty training in preventive medicine or occupational health. Additionally, because heretofore public health has not had a specific journal dedicated to its pedagogy, many potential contributions remain unpublished. Myriad reports about the status of the public health workforce are white papers or government reports and are not converted to publication in peer reviewed literature. In short, the challenges to finding the literature that analyzes and evaluates education for public health professionals is part of the reason that public health has been criticized for its pedagogy, or lack thereof.

## Materials and Methods

To identify critical thinking about the education of public health professionals as reflected in the published literature, a medical librarian conducted a detailed literature search using a sophisticated Boolean logic. Three major data bases were searched: PubMed, Scopus, and Education Full Text. The timeframe was limited to 2000 and forward, recognizing that the education of the professionals for public health practice is a centuries-old endeavor, and a number of seminal articles and reports pre-date this timeframe. The analysis was limited to literature published in peer review journals. White papers, institutional reports and “gray” literature were not included. The search requested articles published in English as the primary language.

The search intentionally did not pursue sub-specialties within public health, such as environmental health, community health education, public health nursing, or nutrition. Sub-specialties, particularly ones that have specific certifications, licenses, or accreditations, might also well have a distinct body of literature on the pedagogy pertaining to that sub-specialty. Similarly, the search did not examine each distinct health professions discipline for articles pertaining to education about public health. If an article appeared in the general search for public health professions pedagogy, it was assumed to have widespread applicability or interest, even if emanating from a sub-specialty of public health or a separate health professions discipline.

The articles were initially categorized using 10 themes generated from a previous pilot study of 110 articles. (These 110 articles were excluded from the retrieved articles.) The retrieved articles were also categorized by type of study: program description, commentary, evaluation, or original research. One of the authors reviewed the abstracts of all articles for (1) relevancy to the study, (2) appropriateness of theme category, (3) type of article. The articles were also examined for date of publication and the publishing journal.

## Results

A total of 576 unique articles were identified from the three sources: PubMed – 478; Scopus – 49; Education Full Text – 49. The 110 articles from the pilot study, relevant to the overall body of literature, were excluded since they had been used to create the initial theme categories. Because the preponderance of articles came from PubMed, and the information available from the other two sources was in different formats, the analysis was limited to those articles published in PubMed. As noted, the abstract for each of the 478 articles was read to confirm appropriateness for the study and for the theme category. Four articles were shifted from one category to another. Fourteen articles were dropped as not directly related to the education of the public health workforce, leaving a total of 464 articles.

Table 1 shows the final breakout by number of articles by theme. Each article was listed under only one theme, although some clearly covered two or more, such as a paper on the competencies of doctoral education or MPH programs in Canada. Most of the theme labels are self-explanatory. The category of “Subject-specific” included articles such as “How to Teach HIV-Prevention Effectively” or “Teaching Maternal and Child Health.” The theme of “Under-represented” derived primarily from efforts to recruit under-represented minority groups into academic ranks through mentoring initiatives.

**Table 1:** **Themes of articles listed in Pubmed pertaining to pedagogy of public health professionals education, 2000–2012**.

Theme	Number of articles
Undergraduate	6
Masters	25
Doctoral	3
Competencies	43
Service learning	51
Workforce	85
Interprofessional	113
International	91
Under-represented	14
Subject-specific	34
Total	464

Table 2 shows the articles by type. The characteristics used to determine the “type” of article related to methodology and genre. Four categories were used:

**Commentary:** articles based on application of concepts, suggestions of theoretical frameworks, analyses without quantifiable data, opinions based on experience and wisdom.
**Program description:** descriptions of a course, curricula, or other teaching approach used primarily by one organization.
**Evaluation:** objective data gathered to evaluate a specific course, curriculum, or teaching approach that had already been implemented.
**Research:** original research conducted to guide the development of a new educational program, such as needs assessment surveys, focus groups, descriptive information compiled across a number of programs.

**Table 2:** **Types of articles related to the pedagogy of public health professionals education, 2000–2012**.

Type of article	Number of articles
Commentary	117
Program description	202
Evaluation	85
Research	60
Total	464

Many articles were descriptions of training programs or case studies of the education offered by a single university. Evaluations of training programs were called out separately, with any other type of quantitative analysis assigned the category of “research.” Commentaries included opinion pieces by thought leaders and analyses of educational trends.

The types of articles were analyzed by theme. Table 3 lists the themes and the types of articles for each theme. Consistently across all themes, Program Descriptions were the most common type of article. Research and Evaluation articles were far fewer.

**Table 3:** **Articles pertaining to the pedagogy of public health professionals education by theme and type**.

Theme	Total number of articles	Program descriptions	Commentary	Training and curricula evaluations	Research
Undergraduate	6	4	2	0	0
Graduate	25	11	8	4	2
Doctoral	3	0	3	0	0
Competencies	43	20	5	9	9
Service learning	51	24	15	10	2
Workforce	85	30	11	28	16
Interdisciplinary	113	59	27	13	14
International	91	43	20	19	9
Under-represented	14	7	3	2	2
Subject-specific	33	4	23	0	6
Total	464	202	117	85	60

Articles within each theme were further analyzed by number of journals. Table 4 shows the total number of discrete journals by theme. (The total is not given as it does not represented an unduplicated count; some journals published articles on more than one theme.) Consistent with the lack of a single journal focused on the pedagogy of public health professionals’ education, articles appeared in a wide array of journals. The average number of articles pertaining to the education of the public health workforce appearing in any given journal over the 12+ year period of the study was a little over two articles per discrete journal over 12 years. To be clear: one journal that published two articles at different points in time would be counted as one journal, not two.

**Table 4:** **Number of journals publishing articles related to the pedagogy of public health professionals education, 2000–2012**.

Theme	Number of articles	Number of discrete journals	Articles per journal
Undergraduate	6	4	1.5
Graduate	25	4	6.25
Doctoral	3	3	1.0
Competencies	43	18	2.4
Service learning	51	22	2.3
Workforce	85	37	2.3
Interprofessional	113	49	2.3
International	91	54	1.6
Under-represented	14	4	3.5
Subject-specific	33	19	1.7
Total	464		

Special topic issues and/or supplements brought together a number of papers on the same issue. Supplements and special issues tended to reflect an educational initiative (usually funded by the US Centers for Disease Control and Prevention), indicating the influence that a dedicated funding initiative can have on education and the need to document the results. Special topics covered themes such as emergency preparedness and the training of veterinarians about public health.

## Discussion

In contrast to the assertion by Frenk and Chen that the field of public health has relatively few articles on its pedagogy, the initial search here identified 686 articles in peer review journals between 2000 and 2012 listed in four different sources (three bibliographic data bases and the pilot study). Publications listed in PubMed (464) were the focus of detailed analysis based on abstract and other information listed in the PubMed data base.

Ten themes of primary content and four types of methods emerged. The themes encompassed articles on content of education (e.g., competencies, subject-specific education), target audience (undergraduate, masters, doctoral, workforce, interprofessional), program descriptions (e.g., courses, curricula, broad initiatives), and others. The types of articles included commentaries and theoretical frameworks, program descriptions, evaluations, and original research. Many articles spanned more than one theme.

The 464 articles appeared in a total of 138 journals, reflecting the breadth of the field and the lack of a single journal focused on the pedagogy of public health. Each theme also had a number of journals, with no theme having a single dominant journal. The number of articles per year varied, but several supplements (by *the Journal of Public Health Management and Practice, the Journal of Veterinary Medicine, and the Journal of the American Public Health Association*) resulted in an increase in papers in a single year.

Before considering implications, the limitations of the study must be recognized. The literature search was limited to one of three widely available data bases of peer review articles. Gray literature pertaining to public health workforce or education of professionals was not combed. The literature search requested articles written in English as the primary language, which likely resulted in the omission of many articles written originally in Spanish, various European, and other languages. The identified literature also pertained primarily to those engaging in public health practice at a professional level, with less attention to support personnel or paraprofessionals, a cadre of public health workforce particularly prevalent in developing nations. Finally, the literature search timeframe was 2000 forward.

Within these limits, the results of the literature search suggest direction for the pedagogy of the public health professional. First, a single conceptual framework – to be practical – must be very broad. Although arguably now recognized as a distinct discipline (5), public health is also inextricably interdisciplinary. This complicates delineating the educational pathway. Education to date has been fragmented, occurring within numerous disciplines and sub-specialties. The outcomes of education have also been diverse and often not clearly articulated, making it difficult to evaluate the effectiveness of the teaching. Where a credential or licensure is the end result, the curriculum and evaluation tend to be highly focused, even to the extent of being siloed. To build a useful body of knowledge about how to teach and what to teach the current and future public health professional workforce, the overarching pedagogical framework must accommodate insights and research from a variety of perspectives that apply across disciplines, credentials, institutions, and nations.

Needs assessment surveys and evaluations of training programs are relatively common. However, original research studies on education of the public health professional workforce and the impact of that education, at least in the published peer review literature, are relatively few compared to descriptive studies and commentaries. This suggests the need for increased rigor in identifying the underlying factual basis to support pedagogical approaches.

The desired outcomes are the starting point for the process of developing an educational program. Although we are aware of numerous reports on the future need for public health workers in the gray literature, very few published studies related education to subsequent practice abilities or career outcomes. A number of needs assessment surveys did ask public health practitioners about their existing or needed competencies, skills, or subject knowledge. Jobs for which licensing or certification are required are more easily related to educational requirements and outcomes. However, because of the varied nature of public health career paths, tying the education to long-term job tasks and career paths is, for the most part, missing in the broad literature.

The funding of special educational initiatives has the potential to result in literature that provides documentation of the rationale for and results of the training program. For example, when the US Centers for Disease Control and Prevention (CDC) became concerned about zoonotic diseases, an emphasis was put on educating veterinarians about public health. This produced curriculum innovations in a number of US universities. The current push toward translation and dissemination of new knowledge no doubt contributed to a desire to publish information about the initiative, and two separate journals ran special issues on veterinary public health education. Similarly, a CDC-funded program to recruit minority researchers to the study of health disparities produced a number of innovative university-based programs, subsequently described in a special issue of a journal. Thus, educational programs and the dissemination of information about them in formal peer review literature can be directly affected by funding sources and their expectations of translation and dissemination.

## Conclusion

The literature pertaining to the pedagogy for public health professionals reflects the status of the field, as well as its approach to education. The analysis of the literature reported here reinforces the following:

- Education of the professional workforce for public health practice is distinct from educating the lay public to engage in positive health practices, although there is overlap.
- Those teaching professionals for the field of public health clearly think about what they teach, how they teach, and what approaches are most effective for which purposes.
- Public health is a highly interdisciplinary field, with education incorporated into the education of various health professions as well as a distinct academic discipline of public health.
- Within public health, some sub-specialties are more advanced in their pedagogy than others. This is often tied to specific licensure or certification and accreditation by external accrediting bodies.
- As public health grows as a focus of education at the university undergraduate level, the pedagogy of public health should be used to guide instruction, curriculum articulation, career planning, and practice expectations. Published articles on undergraduate public health education tend to be unrelated to articles on general public health workforce education, graduate, or sub-specialty education.
- Practice is clearly a desired outcome yet it is difficult to measure the impact of education over time; additional work on evaluating public health professionals’ education is warranted. Licensure and certification data bases and content expectations offer one way to evaluate the effects of formal education.
- Relating public health education across national boundaries and education systems is complex given the diversity of public health education within a given country. More information about how public health education is approached in individual countries, perhaps shared through open access literature, would provide a basis of knowledge for sharing educational content and platforms across nations to achieve consistency in the education of a global public health workforce.
- A search term or way of identifying literature on education for public health professionals will facilitate access to peer reviewed information that can inform the pedagogy of public health professionals’ education.

## Conflict of Interest Statement

The authors declare that the research was conducted in the absence of any commercial or financial relationships that could be construed as a potential conflict of interest.
